# Cultivar and Year Rather than Agricultural Practices Affect Primary and Secondary Metabolites in Apple Fruit

**DOI:** 10.1371/journal.pone.0141916

**Published:** 2015-11-30

**Authors:** Carine Le Bourvellec, Sylvie Bureau, Catherine M. G. C. Renard, Daniel Plenet, Hélène Gautier, Line Touloumet, Thierry Girard, Sylvaine Simon

**Affiliations:** 1 UMR408 Sécurité et Qualité des Produits d'Origine Végétale, Institut National de la Recherche Agronomique, Avignon, France; 2 UMR Sécurité et Qualité des Produits d'Origine Végétale, Université d'Avignon et des Pays de Vaucluse, Avignon, France; 3 UR1115 Plantes et Systèmes de culture Horticoles, Institut National de la Recherche Agronomique, Avignon, France; 4 UE695 Recherches Intégrées, Domaine de Gotheron, Institut National de la Recherche Agronomique, Saint-Marcel lès-Valence, France; Universidade Federal de Viçosa, BRAZIL

## Abstract

Many biotic and abiotic parameters affect the metabolites involved in the organoleptic and health value of fruits. It is therefore important to understand how the growers' decisions for cultivar and orchard management can affect the fruit composition. Practices, cultivars and/or year all might participate to determine fruit composition. To hierarchize these factors, fruit weight, dry matter, soluble solids contents, titratable acidity, individual sugars and organics acids, and phenolics were measured in three apple cultivars (‘Ariane’, ‘Melrose’ and ‘Smoothee’) managed under organic, low-input and conventional management. Apples were harvested at commercial maturity in the orchards of the cropping system experiment BioREco at INRA Gotheron (Drôme, 26) over the course of three years (2011, 2012 and 2013). The main factors affecting primary and secondary metabolites, in both apple skin and flesh, were by far the cultivar and the yearly conditions, while the management system had a very limited effect. When considering the three cultivars and the year 2011 to investigate the effect of the management system *per se*, only few compounds differed significantly between the three systems and in particular the total phenolic content did not differ significantly between systems. Finally, when considering orchards grown in the same pedoclimatic conditions and of the same age, instead of the usual organic *vs*. conventional comparison, the effect of the management system on the apple fruit quality (Fruit weight, dry matter, soluble solids content, titratable acidity, individual sugars, organic acids, and phenolics) was very limited to non-significant. The main factors of variation were the cultivar and the year of cropping rather than the cropping system. More generally, as each management system (e.g. conventional, organic…) encompasses a great variability of practices, this highlights the importance of accurately documenting orchard practices and design beside the generic type of management in such studies.

## Introduction

Organically grown trees, without synthetic inputs, are more exposed to environmental constraint (pest or disease occurrence, nitrogen availability) than orchards using synthetic inputs. Since environmental factors play a role in a plant’s production of secondary metabolites, in response to stresses, it is often assumed that organic agriculture would increase the biosynthesis of secondary metabolites (such as phenolics) in plant and fruit [1]. Flavonoids are produced as a defense mechanism that can be influenced by nutrient deficiencies such as the lack of nitrogen in the soil [2]. However, one of the pitfalls of the studies on the subject lies in the characterization of the experimented production systems: each production system, whether organic or conventional, encompasses a great variability of practices under 'organic' or 'conventional' denomination. [3] Identified the crop load and the nitrogen level as key elements responsible for the synthesis of total phenolics and sugar content of peaches beyond the production system *sensu stricto*. For apple (*Malus* x *domestica*), the effect of crop load on the fruit phenolic content has been demonstrated in some studies [4] but not for all [5–6]. Moreover, it is sometimes difficult to obtain fruit grown under different management systems in identical environmental conditions: sets of paired farms [7–8] are often used, with limits in terms of variability of external factors (e.g., soil, climate) or orchard design (e.g., age, rootstock, training and management of trees). In most studies also only one harvest year is taken into account, giving little information on preplanting conditions (e.g., tillage, cover-crop, previous crop, etc.) and providing almost no information on the period the orchard has been under a given management system prior to the study.

Cropping system experiments are new approaches that overcome some of these biases. Indeed, according to context, constraints and main objectives (e.g., weed control with no herbicides in annual crops), cropping system experiments permit to iteratively design, test and evaluate cropping systems through multi-criteria and multi-year assessment [9]: generally tested in large fields or plots to account for the studied processes (especially related to pest pressure), the experimented systems are production units accurately documented for their environmental conditions, design and practices. In fruit tree production, an 'orchard system' can be seen as a cultural unit designed and managed identically to meet one (or more) objective(s), with the implementation within time and/or space of a coherent set of methods to achieve these goals [10].

Apples are the most consumed fresh fruit in France (about 20 kg per person per year; [11]) and in Europe [12] and are an important source of phenolics in the diet [13]. Soluble sugars and organic acids are important components of fruit taste. If the visual and organoleptic qualities of this fruit are important criteria for the consumer, health value is also an important asset: for a low calorie intake, apple is rich in fibers (including pectins) and phenolics [14]. Epidemiological studies have revealed an inverse correlation between the consumption of apples and apple flavonoids level and coronary mortality [15–16].

Qualitative and quantitative phenolics, individual sugar, and organic acids composition is well-documented in apple [14, 17–24]. The content of phenolics, sugars and organic acids in apple are influenced by several biotic and abiotic factors such as cultivar [18, 20, 22–23, 25–26], rootstock [27], climate [7], temperature, light, and water availability modulated by irrigation [28]. The available data on the effect of the orchard practices and management system on phenolic content of apple give controversial results. Most of the studies compare organically and conventionally grown apples. Phenolic content can be significantly higher in apples from organic production [29]. The effect of climate [7] or cultivar [30] can be higher than the management system effect *per se*, or similar to the management system [8, 31]. Phenolic content can also be significantly higher in apples under integrated production (i.e., management system aiming to rely on ecosystem services and promoting the use of alternative methods to chemicals) compared to organic [32, 33]. Such differences may be due to studied cultivars, growing conditions and/or methods of sampling and analysis, and it is therefore difficult to have a clear idea of the relative effect of the management system on apple phenolic content.

The specific objective of this work was to study over three growing seasons (2011–2013) the fruit quality and nutritional parameters of apples grown since 2005 under conventional, low-input and organic management systems in the same cropping system experiment, i.e. in the same environmental conditions for soil and climate. Moreover, as these management systems were each planted with three cultivars differing in scab susceptibility, the trial resulted in nine ‘management × cultivar’ apple orchard systems [34], that allowed to assess the effect of both the cultivar and the management system on the fruit primary and secondary metabolites.

## Materials and Methods

### Standards and chemicals

Acetonitrile of HPLC grade and acetic acid were from Fischer Scientific (Pittsburgh, PA, USA). 5-O-caffeoylquinic acid, (+)-catechin, (-)-epicatechin, sucrose, glucose, fructose, citric acid and toluene-α-thiol were from Sigma Aldrich (Darmstadt, Germany). Phloretin, *p*-coumaric acid, quercetin and cyanidin-3-O-galactoside were obtained from Extrasynthese (Lyon, France). Phloridzin was obtained from Fluka (Buchs, Switzerland). Malic acid was obtained from R-Biopharm (Darmstadt, Germany).

### Cropping system

The experimental orchards were planted in January 2005 at the National Institute for Agricultural Research (INRA) Gotheron experimental station in the middle Rhone valley (South-East France, 44°58′33″ N, 4°55′45″ E). The design and management practices of the system experiment are described in Simon et al. (2011) [34] and summarized in Table 1. Briefly, three management systems were combined with three cultivars in nine orchard systems of 0.36 ha each planted at the same site in similar environmental conditions:

- Conventional: The aim is input efficiency; synthetic pesticides are the main tool to control pests, diseases and weeds, and mineral fertilizers are exclusively used;
- Low-input: The aim is to combine various substitution methods to avoid or limit pesticide use, and both organic and mineral fertilizers are used;
- Organic: The aim is to avoid direct measures against pests and diseases within the framework of European and national rules (EEC 834/2007 and applications) and national organic rules, i.e. synthetic inputs are banned.

**Table 1:** Main practices used in the three experimented management systems in the 2011–2013 period.

Management system	Conventional	Low-input	Organic
Pest and disease management	Sanitation practices, mating disruption (since 2012), chemical and microbiological insecticides, decision support systems	Sanitation practices, mating disruption, (micro)biological insecticides, kaolin, decision support systems	Sanitation practices, mating disruption, (micro)biological insecticides, kaolin, decision support systems & no synthetic pesticides
Within-row weed management	Chemical	Mechanical	Mechanical
Between-row management	Mechanical schredding	Mechanical schredding	Mechanical schredding
Type of fertilizer, total yearly nitrogen availability (kg ha^-1^)	Mineral, 75 kg ha^-1^	Mix of organic ^a^ and mineral, 70 kg ha^-1^	Organic ^a^, 80 kg ha^-1^
Irrigation	Dripped irrigation, water balance	Dripped irrigation, water balance	Dripped irrigation, water balance
Tree training	Vertical axis, centrifugal training	Vertical axis, centrifugal training	Vertical axis, centrifugal training
Fruit load (fruit cm^-2^ trunk cross section area (TCSA))	6 fruits cm^-2^ TCSA	6 fruits cm^-2^ TCSA	4 fruits cm^-2^ TCSA

^**a**^ Since organic nitrogen from compost is not directly available for the trees, nitrogen availability was estimated to be 45% of the total nitrogen content in the year when the compost is applied, then 25% and 15% in the following years.

In each management system, three cultivars differing in scab susceptibility (scab is a major apple fungus disease due to *Venturia inaequalis*) were planted grafted onto PI80 (INFEL 6275^®^) rootstock at 1000 trees ha^-1^:

- Smoothee (INFEL^®^ 2832) is a mutant of Golden Delicious, here referred to as ‘Smoothee’, and is a disease-susceptible cultivar;
- ‘Ariane’ (INFEL^®^ 6407) is resistant to the common strains of scab;
- ‘Melrose’ (INFEL^®^ 2643) is a low-susceptibility cultivar for diseases.

Consequently, the three management systems differed for protection (management of pests, diseases and weeds), fertilization (type of fertilizers) and fruit load which is adjusted to the orchard yield potential and was also lowered in organic farming to limit alternate bearing, but they were under similar management for all other practices (Table 1).

Agronomic results (tree vigor, quantity and quality of yield) can vary according management systems, cultivars and years. To highlight such variability, the agronomic performances of the cultivar Ariane are presented for the year 2011 (S1 Table): tree vigor estimated by the trunk cross section area (TCSA) is similar among management systems; in contrast, yield is lower in organic farming, due to a lower fruit load and smaller fruits; fruit damage due to pests tends to be higher in organic farming.

### Plant material and sample preparation

For each study plot, 30 fruits were collected just before the main harvest on trees with a normal load (no tree displaying alternate bearing, no overloaded tree) and located in the 4 center lines of each plot. The fruits were randomly selected from ground level, among the most colorful and deemed fit to be picked with 15 East-exposed and 15 West-exposed fruits. They were identified and sent immediately to the laboratory for analysis. To avoid impact of inter-person variation in fruit choice, all harvests were carried out by the same person for all three years.

Three triplicates of 10 apples were constituted. Each apple was peeled and cored and then divided as described in Le Bourvellec et al. (2011) [14]. Apples were divided in two containers A and B. The containers A were freeze-dried and used for phenolic characterization. Containers B were frozen and used for infrared and sugars and acids characterization.

Physico-chemical analyses were carried out separately on the pulp and the skin, for three main reasons:

- the two tissues have very different compositions;
- skin, being directly exposed to the outer conditions, might be supposed to be more variable with the growing conditions and agronomic practices, therefore this might increase possibility to detect weak effects;
- many people eat apples peeled, therefore nutritional relevancy needs separate analyses.

### Analysis methods

Total Soluble Solids (TSS) were determined with a digital refractometer (PR-101 ATAGO, Norfolk, VA, USA) and expressed in °Brix at 20°C. Titratable acidity (TA) was determined by titration up to pH 8.1 with 0.1 mol/L NaOH and expressed in mmol H^+^ kg^-1^ fresh weight (FW) using an autotitrator (Methrom, Herisau, Switzerland). Sugars (glucose, fructose, sucrose and sorbitol) and organic acids (malic acid and citric acid) were quantified using colorimetric-enzymatic methods (Boehringer Mannheim Co., Mannhein, Germany) and expressed in g kg^-1^ FW. These measurements were performed with a SAFAS flx-Xenius XM spectrofluorimeter (SAFAS, Monaco).

Phenolics were measured by high-performance liquid chromatography (HPLC)-diode array detection (DAD) after thioacidolysis as described by Le Bourvellec et al. (2011) [14]. Procyanidins were characterized by thioacidolysis to determine subunit composition, average molecular mass and the average degree of polymerization (mDP). The mDP of procyanidins was calculated as the molar ratio of all the flavan-3-ol units (thioether adducts plus terminal units) to (-)-epicatechin and (+)-catechin corresponding to terminal units. HPLC-DAD analyses of methanolic extracts which were not submitted to thioacidolysis were performed to assay separately monomeric catechins and procyanidins. Analyses were performed using an Ultra Fast Liquid Chromatography Shimadzu Prominence system (Kyoto, Japan) including two pumps LC-20AD Prominence liquid chromatograph UFLC, a DGU-20A5 Prominence degasser, a SIL-20ACHT Prominence autosampler, a CTO-20AC Prominence column oven, a SPD-M20A Prominence diode array detector, a CBM-20A Prominence communication bus module and controlled by a LC Solution software (Shimadzu, Kyoto, Japan). Separations were achieved in Le Bourvellec et al. (2011) [14] conditions. Individual compounds were quantified in mg kg^-1^ FW by comparisons with external standards at 280 nm for (+)-catechin, (-)-epicatechin, phloretin xyloglucoside (quantified as phloretin), phloridzin, (-)-epicatechin benzyl thioether (quantified as (-)-epicatechin); at 320 nm for 5-O-caffeoylquinic acid, *p*-coumaroylquinic acid (quantified as *p*-coumaric acid) and their methylated derivatives obtained during thioacidolysis reaction quantified as their respective non-methylated equivalents, at 350 nm for quercetin glycosides (quantified as quercetin) and at 540 nm for cyanidin glycosides (quantified as cyanidin-3-O-galactoside).

### Infrared spectroscopy

As described in [35], mid-infrared spectra were collected at 23°C with a Bruker Tensor 27 FTIR spectrometer (Wissembourg, France) equipped with a horizontal attenuated total reflectance (ATR) sampling accessory and deuterated triglycine sulphate (DTGS) detector. The homogenized (fruit purees) samples were placed at the surface of the zinc selenide crystal providing six internal reflections into the samples. The sample consistency, a thick liquid, allowed a good contact between sample and crystal and did not require pressing. The samples were scanned at wavenumbers from 4000 cm^-1^ to 650 cm^-1^, and corrected against the background spectrum of air. The spectrum of each sample was the average of 32 scans. The crystal was cleaned between measurements with deionized water and dried with lint-free tissue. Instrument control and spectral collection were performed using OPUS software (version 4.0 Bruker, France) supplied by the manufacturer.

### Statistical analysis

Results are reported as mean values ± standard deviation (SD). PCA and (3 ways and 2-ways) ANOVA (considering cultivar, management system and year) were performed on physico-chemical data using the Excelstat package of Microsoft Excel. Spectral preprocessing and multivariate data analysis were performed with Matlab 7.5 (Mathworks Inc. Natick, MA) software using SAISIR package [36]. The absorption band around 2400 cm^-1^, due to carbon dioxide, was discarded. Spectra were systematically pre-treated by standard normal variate correction (SNV).

## Results

### Apple metabolite contents and fruit characteristics

Table 2 presents the range of variation of dry matter (%) and of individual phenolics, individual sugars, organic acids, total soluble solids, titratable acidity of apple pulp and skin. The whole dataset is presented in supporting information (S2 and S3 Tables).

**Table 2:** Range of variation of dry matter (%), total soluble solid contents (°Brix), individual sugars (g kg^-1^ FW), titratable acidity (mmol H+ kg^-1^ FW), organic acids (g kg^-1^ FW) and phenolics (mg kg^-1^ FW) of apple samples from 3 management systems x 3 cultivars in 3 years.

	Apple tissues and samples			
	Skin		Pulp	
	Min.	Max.	Min	Max
Dry matter	18.5	24.6	13.2	18.7
	MC11	AO12	ML11	AO12
Total soluble solids	11.2	18.7	10.8	15.5
	MC12	AO12	MC12	AO12
Glucose	12.1	26.5	9.6	26.4
	AC13	MO11	AC13	MC11
Fructose	40.3	65.4	53.3	90.6
	MO13	MC13	AL13	MO13
Sucrose	16.0	51.5	10.5	64.9
	ML11	AO13	MO11	AL13
Sorbitol	2.2	10.2	1.6	11.4
	ML13	AO12	SC13	AO12
Titratable acidity	36.1	114.6	44.4	115.9
	MO11	AO12	ML13	AO11
Malic acid	3.1	9.0	3.8	9.8
	ML13	AO11	MC13	AO12
Citric acid	nd	0.36	nd	0.47
		AL12		AL12
Sum of phenolics	2254	3887	400	727
	SL12	AL13	MO11	AO13
Procyanidins	1315	2580	257	500
	ML11	AL13	SL13	MC12
mDP	5.2	8.2	4.2	9.2
	SO12	MO13	SC12	SC11
Flavan-3-ols				
(-)-epicatechin	110	260	34	64
	SC11	AL13	AO12	ML13
(+)-catechin	10	39	8	23
	SC11	AL13	AO11	AC11
Dihydrochalcones				
phloridzin	32	110	7	20
	AC11	MO13	MC11	AO13
phloretin-2-O-xyloglucoside	15	51	3	7
	SO11	MC12	SC11	MO13
Hydroxycinnamic acids				
5CQA	12	174	47	215
	MC11	AL13	MC11	AO13
*p*CoQA	1	6	3	9
	MC11	AO12	SC11	MC12
Flavonols	294	792	nd	nd
	AC13	SO12		
Anthocyanins	45	402	nd	nd
	MC12	AO11		

The code under the value indicates the precise sample: the first letter for the cultivar (M: Melrose, S: Smoothee, A: Ariane), the second for the management system (C: conventional, O: organic, L: low-input), and the numbers designate the year (2011, 2012, 2013). mDP: average degree of polymerization of procyanidins, 5CQA: 5-O-caffeoylquinic acid, pCoQA: p-coumaroylquinic acid.

The average fruit weight of the apples ranged 132 to 233 g and dry matter from 18.5 to 24.6% in the skin and from 13.2 to 18.7% in the pulp.

Sugars and organic acids define the taste balance and, together with volatile compounds, the flavor of apples. Total soluble solids content ranged 11.2 to 18.7 g kg^-1^ FW in the skin and 10.8 to 15.5 g kg^-1^ FW in the pulp. Sucrose and fructose were the major sugars found in the skin and in the pulp (Table 2), while glucose and sorbitol were relatively minor components. Titratable acidity ranged 36.1 to 114.6 mmol H^+^ kg^-1^ FW in the skin and 44.4 to 115.9 mmol H^+^ kg^-1^ FW in the pulp. Malic acid was the prevalent organic acid, and citric acid was only quantified in minor amounts.

Five phenolic classes with a total of sixteen identified individual compounds were quantified in the pulp and in the skin of apple (Table 2). For all cultivars, the sum of phenolics content of the pulp was significantly lower than in the skin. Among the five major groups, the procyanidins (PC), flavan-3-ol polymers or condensed tannins, were the predominant class both in the pulp and in the skin, accounting for over 65% of the total phenolics. Flavan-3-ol monomers detected in the pulp and in the skin were (-)-epicatechin and (+)-catechin, the former being predominant. Hydroxycinnamic acids represented the second phenolic class in the pulp with up to 20% of total phenolics, while in the skin they accounted for less than 3%. The main compound in the hydroxycinnamic acids class was 5-O-caffeoylquinic acid (5CQA) followed by *p*-coumaroylquinic acid (*p*CoQA). Dihydrochalcones (phloridzin and phloretin-2-O-xyloglucoside) were a minor group, accounting for an average of 3% and 3.5% of the total phenolic in the pulp and in the skin, respectively. Skin differed from pulp by the additional presence of flavonols (quercetin glycosides) and, in red-skinned apples, of anthocyans (cyanidin glycosides). Flavonols represented the second highest class in apple skin, with more than 16% of its sum of phenolics. Six quercetin glycosides were found and quantified in quercetin equivalent: quercetin-3-O-galactoside> quercetin-3-O-arabinopyranoside> quercetin-3-O-rhamnoside> quercetin-3-O-glucoside> quercetin-3-O-xyloside> quercetin-3-O-rutinoside. Cyanidin glycosides accounted for 1–12% of total phenolics in the skin of ‘Ariane’ and ‘Melrose’. They were represented by cyanidin-3-O-galactoside and cyanidin-3-O-pentoside in ‘Ariane’ cultivar while, in ‘Melrose’ cultivar, only cyanidin-3-O-galactoside was detected.

### Typology of the fruit based on mid-infrared spectra

**Fig 1:**
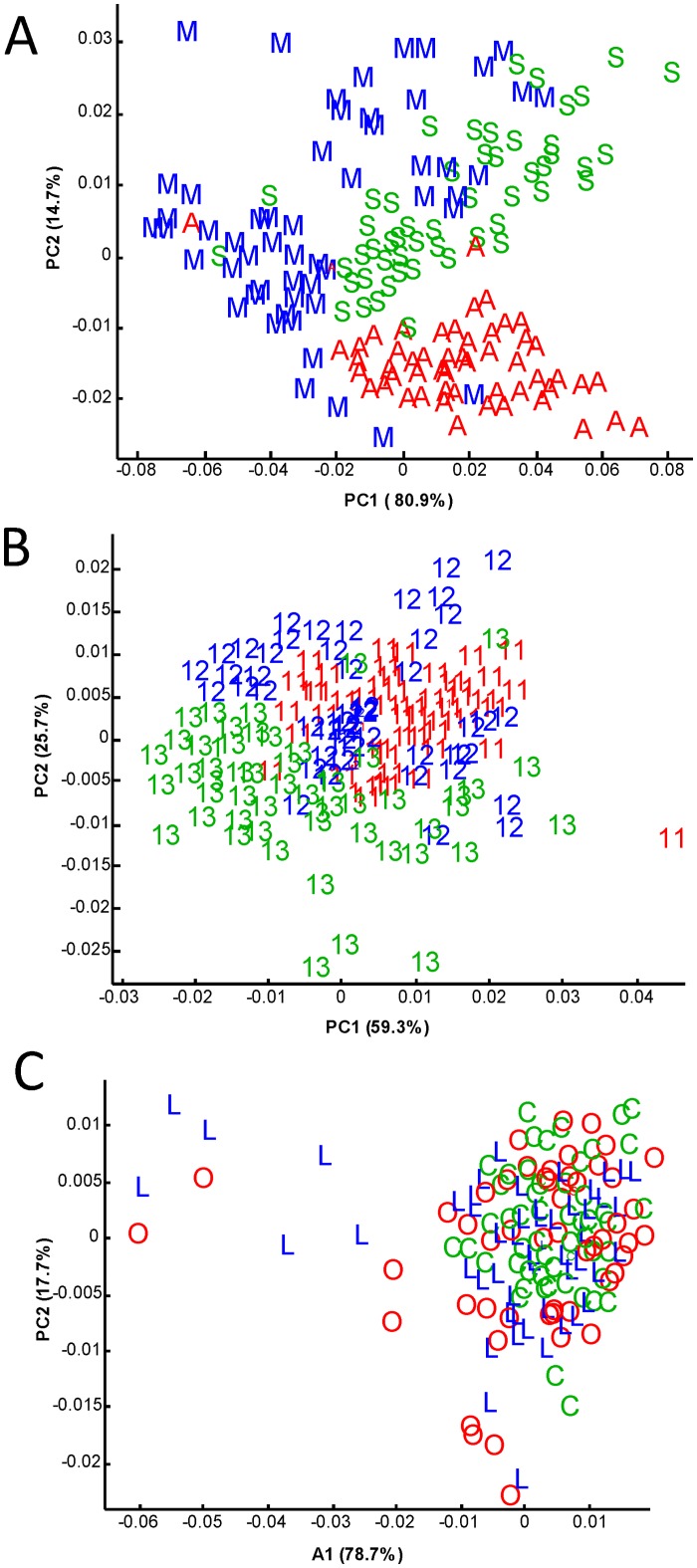
PCA results on mid-infrared spectral data of apple skin and pulp. A: spectral area between 1500–1000 cm^-1^, B: spectral area between 2000–1500 cm^-1^ and C: spectral area between 3000–2500 cm^-1^. The code corresponds to the cultivar (M: Melrose, S: Smoothee, A: Ariane), to the management system (C: conventional, O: organic, L: low-input) and to the year 2011, 2012 and 2013.

In the fingerprint region (1500 and 900 cm^-1^), i.e. the absorption range of different molecular vibrations such as OH-bending, C-O and C-C stretching, the MIR spectra differ in absorbance in relation to the variability of the apples composition [23]. This spectral range also contains quantitative information a.o. about the sugars, organic acids and phenolics present, as demonstrated in Bureau et al. (2012) [23]. They integrate the composition of the apple samples, and are a suitable signal for to evaluate the variability within such sample sets. ANOVA performed on spectral data allowed to classify the studied factors according to their global effect. The Fisher values (F) decreased in the order: cultivar (F = 120) > year (F = 55) > system (F = 7) (results not shown). The effect of ‘cultivar’ was significantly higher than that of ‘year’, itself much higher than the effect of ‘system’. ANOVA also identified specific spectral areas showing variation for each factor. The specific spectral areas were 1500–1000 cm^-1^ for cultivar, 2000–1500 cm^-1^ for year and 3000–2500 cm^-1^ for system. These areas were used to discriminate the apple samples using principal component analyses (PCA, only the two first principal components (PC)) shown in Fig 1. The three cultivars appeared relatively well discriminated by PCA using the most discriminating spectral area, 1500–1000 cm^-1^ (Fig 1A). As regards the year, 2011 and 2013 were fairly well discriminated by PCA using the spectral area 2000–1500 cm^-1^, forming two separate clusters. In contrast, 2012 was distributed in three clusters that partly overlapped the 2011 and 2013 years. The three management systems completely overlapped, even when the most discriminating spectral area was used, 3000–2500 cm^-1^ (Fig 1C). Whereas the variability given by cultivars and years was large enough to be observed in mid-infrared, differences due to the management systems were too small to discriminate the samples by their mid-infrared spectra.

### Apple metabolites and fruit characteristics as affected by cultivar, cropping year and management system

**Fig 2:**
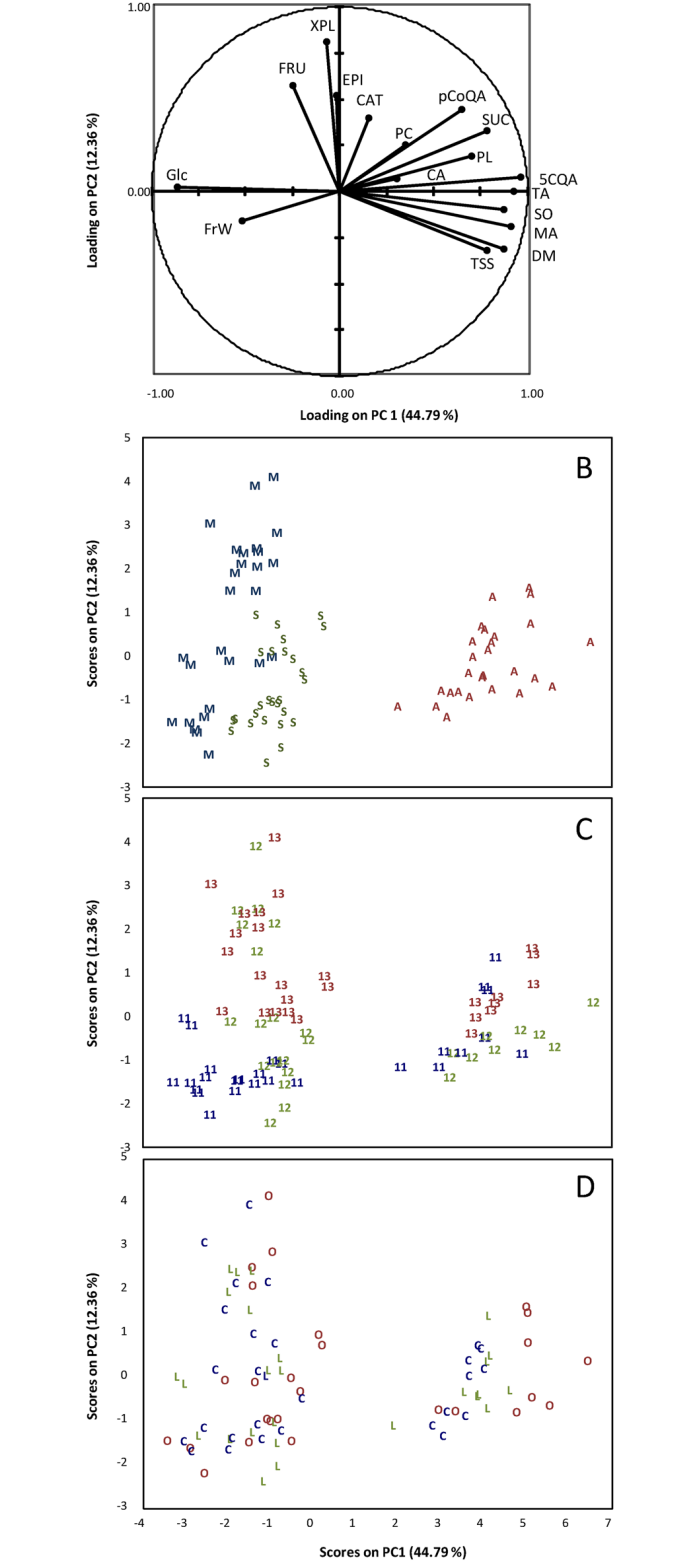
PCA results on individual sugars, organic acids, titratable acidity, dry matter, fruit weight, phenolics and total soluble solids of apple pulp. A: Correlation circle of variables loadings on PC1 and PC2. B: Sample map of scores on PC1 and PC2 as function of the cultivar. C: Sample map of scores on PC1 and PC2 as function of the year. D: Sample map of scores on PC1 and PC2 as function of the management system. The code corresponds to the cultivar (M: Melrose, S: Smoothee, A: Ariane), to the management system C: conventional, O: organic, L: low-input), and to the year (2011, 2012 and 2013).

**Fig 3:**
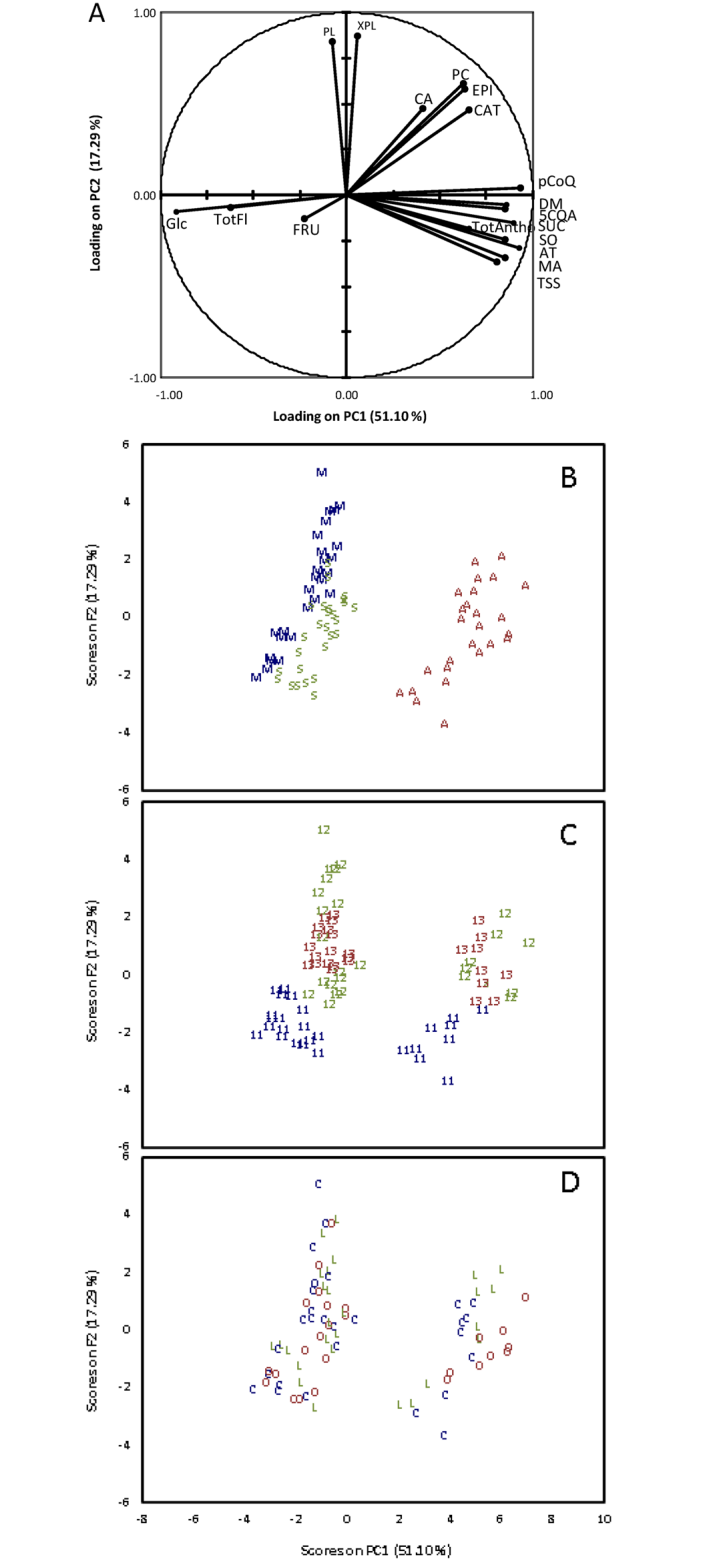
PCA results on individual sugars, organic acids, titratable acidity, dry matter, phenolics and total soluble solids of the skin. A: Scatter plot of loadings on PC1 and PC2. B: Sample map of scores on PC1 and PC2 as function of the cultivar. C: sample map of scores on PC1 and PC2 as function of the year. D: Sample map of scores on PC1 and PC2 as function of the year. The code corresponds to the cultivar (M: Melrose, S: Smoothee, A: Ariane), to the management system C: conventional, O: organic, L: low-input), and to the year (2011, 2012 and 2013).

ANOVA and PCA were applied to all chemical and physico-chemical characteristics of apple pulp and skin (Figs 2 and 3, Tables 3 and 4).

**Table 3:** Fisher’s F-values and significance associated with ANOVAs (management system, cultivar, year) performed on apple pulp metabolites, dry matter, and fruit weight.

	FrW	DM	TSS	SUC	GLC	FRU	SO	TA	MA	CA	CAT	EPI	PC	mDP	XPL	PL	5CQA	*p*CoQA	Tot
C	212.8	774.9	158.8	252.3	242.2	14.1	331.4	713.7	565.4	7.2	7.2	8.2	10.7	3.	235	51.1	875.6	168.2	76.9
	***	***	***	***	***	***	***	***	***	*	*	**	**	*	***	***	***	***	***
M	134.9	17.5	0.2	3.3	5.2	2.2	2.4	12.9	17.2	4.0	16.6	4.5	1.4	0.1	14.9	11.0	1.0	4.3	0.8
	***	***	ns	*	*	ns	ns	***	***	*	***	*	ns	ns	***	***	ns	*	ns
Y	143.8	39.7	18.4	167.2	16.9	7.6	11.6	0.4	28.3	43.4	11.5	6.7	6.5	25.4	70.8	29.5	82.3	57.8	0.7
	***	***	***	***	***	*	***	ns	***	***	***	*	*	***	***	***	***	***	ns
CxM	19.1	6.7	0.57	0.4	3.2	1.8	2.5	1.6	5.8	1.4	3.1	1.8	8.4	0.6	2.2	4.6	5.4	1.7	6.8
	***	***	ns	ns	*	ns	ns	ns	***	ns	*	ns	***	ns	ns	*	**	ns	**
CxY	89.8	0.6	4.6	14.9	7.7	6.7	15.1	10.4	6.3	5.2	4.4	8.3	5.3	5.7	10.5	0.9	0.9	17.8	6.2
	***	ns	*	***	***	***	***	***	***	*	*	***	*	**	***	ns	ns	***	**
MxY	1.1	2.4	2.7	4.5	3.3	1.5	3.2	4.0	3.0	2.3	6.8	1.0	3.7	3.0	0.9	0.7	1.4	7.0	3.3
	ns	ns	*	*	*	ns	*	*	*	ns	**	ns	*	*	ns	ns	ns	***	*
CxMxY	29.4	5.6	3.8	1.3	2.3	2.9	3.2	2.3	2.9	1.2	1.8	1.6	1.3	0.4	5.1	1.4	1.5	3.2	1.2
	***	***	*	ns	*	*	*	*	*	ns	ns	ns	ns	ns	***	ns	ns	*	ns

C: Cultivar, M: Management system, Y: Year, FrW: Fruit Weight, DM: Dry Matter, TSS: Total Soluble Solids, SUC: sucrose, GLC: glucose, FRU: Fructose, SO: Sorbitol, TA: Titratable Acidity, MA: Malic Acid, CA: Citric Acid, CAT: (+)-catechin, EPI: (-)-epicatechin, PC: procyanidins, mDP: average degree of polymerization of procyanidins, XPL: phloretin-2-O-xyloglucoside, PL: phloridzin, 5CQA: 5-O-caffeoylquinic acid, *p*CoQA: *p*-coumaroylquinic acid, Tot: sum of phenolics, ns: non significant.

*: significant at P ≤0.05

**: significant at P ≤0.01

***: significant at P ≤0.001

**Table 4:** Fisher’s F-values and significance associated with ANOVAs (management system, cultivar, year) performed on apple skin metabolites and dry matter.

	DM	TSS	SUC	GLC	FRU	SO	TA	MA	CA	CAT	EPI	PC	mDP	XPL	PL	5CQA	pCoQA	TotFl	TotA	Tot
C.	256.1	179.9	404.4	337.8	7.1	612.4	1188.1	682.3	15.6	115.8	91.3	59.6	12.7	18.0	24.9	1641.4	453.6	36.1	395.9	60.7
	***	***	***	***	*	***	***	***	***	***	***	***	***	***	***	***	***	***	***	***
M.	8.5	6.7	4.6	3.6	0.1	48.5	16.0	23.6	0.6	2.2	6.6	7.8	2.1	4.1	11.1	8.6	2.4	3.3	3.4	2.2
	**	*	*	*	ns	***	***	***	ns	ns	*	*	ns	*	***	**	ns	*	*	ns
Y.	57.8	4.3	33.7	47.2	11.1	28.1	7.8	3.5	41.5	228.2	55.5	35.0	31.3	74.5	92.5	477.0	75.5	16.5	26.8	15.9
	***	*	***	***	***	***	*	*	***	***	***	***	***	***	***	***	***	***	***	***
CxM	11.8	3.1	3.6	7.9	1.8	3.2	10.6	2.4	0.3	1.7	1.9	2.3	0.7	3.0	2.2	6.0	3.2	1.5	1.5	1.6
	***	*	*	***	ns	*	***	ns	ns	ns	ns	*	ns	*	ns	**	*	ns	ns	ns
CxY	7.1	15.4	7.2	7.9	1.5	31.4	0.6	12.0	10.0	10.9	14.8	11.7	6.6	8.9	4.8	18.8	1.0	6.9	8.5	8.0
	**	***	***	***	ns	***	ns	***	***	***	***	***	**	***	*	***	ns	**	***	***
MxY	2.9	1.0	2.0	0.4	0.7	3.7	0.5	0.9	0.9	7.1	6.2	2.0	2.9	0.8	1.0	0.7	2.3	1.9	4.3	3.3
	*	ns	ns	ns	ns	*	ns	ns	ns	**	**	ns	*	ns	ns	ns	ns	ns	*	*
CxMxY	3.7	2.2	4.5	5.2	6.9	9.3	2.1	1.7	0.6	5.0	3.3	1.6	1.4	1.6	2.7	2.8	2.1	1.0	1.4	1.1
	*	ns	**	***	***	***	ns	ns	ns	**	*	ns	ns	ns	*	*	ns	ns	ns	ns

C: Cultivar, M: Management system, Y: Year, DM: Dry Matter, TSS: Total Soluble Solids, SUC: sucrose, GLC: glucose, FRU: Fructose, SO: Sorbitol, TA: Titratable Acidity, MA: Malic Acid, CA: Citric Acid, CAT: (+)-catechin, EPI: (-)-epicatechin, PC: procyanidins, mDP: average degree of polymerization of procyanidins, XPL: phloretin-2-O-xyloglucoside, PL: phloridzin, 5CQA: 5-O-caffeoylquinic acid, pCoQA: p-coumaroylquinic acid, TotalFl: sum of flavonols, TotalA: sum of anthocyanins, Tot: sum of phenolics, ns: non significant.

*: significant at P ≤0.05

**: significant at P ≤0.01

***: significant at P ≤0.001

ANOVAs (Table 3) and PCA (Fig 2) were performed on the chemical and physico-chemical characteristics of the pulp. For all variables the Fisher’s F-values decreased in the order: cultivar > year > management system, except for titratable acidity, (+)-catechin and total phenolic content, for which the order was cultivar > management system > year and for citric acid and mDP, for which the order was year > cultivar > management system.

In the PCA analysis of the chemical and physico-chemical characteristics of the pulp the two first axes (PC1 and PC2) explained more than 57% of the total variance and the following contributions were very low. The first PC-score (PC1) contributed 45% of the total variance and had a high contribution of 5-O-caffeoylquinic acid, titratable acidity, sorbitol and malic acid in the positive direction and of glucose and fruit weight in the negative direction. The second PC-score (PC2) contributed 12% of the total variance and had a high contribution of phloretin-2-O-xyloglucoside variable in the positive direction. Ariane, Melrose and Smoothee cultivars appeared in different zones of the sample map (Fig 2B). Ariane was clearly discriminated from the two others cultivars on PC1. For ‘Melrose’ and ‘Smoothee’, there was some clustering, however the points were more scattered and two ‘Melrose’ samples overlapped the ‘Smoothee’ samples. ANOVA confirmed these representation of variability since the cultivar effect was always the major effect for all the variables (Table 3). This might suggest that there were significant quantitative differences in the compositional profiles of the three cultivars. ‘Ariane’ samples representation in the positive part of the PC1 was explained by its higher contents in 5-O-caffeoylquinic acid, malic acid, sucrose and higher titratable acidity than the other two cultivars but it presented also lower glucose content and fruit weight. ‘Melrose’ samples position was explained by higher fruit weight and glucose content (Fig 2A and S2 Table). Consistently with ANOVA (Table 3) the inter-annual (Fig 2C) differences were less clearly represented than the cultivar differences. As with spectral data (Fig 1B), 2011 and 2013 were fairly well discriminated forming two separate clusters. In contrast, 2012 was distributed in clusters that partly overlapped the 2011 and 2013 years. While sample discrimination was possible according to the cultivar and less clearly to the year according to PC1 and PC2, it was not possible to discriminate samples based on the management system due to the overlapping of all the samples (Fig 2D).

For the skin (Table 4) the Fisher’s F-values of management system were always the lowest for all variables, except for total soluble solids, sorbitol, titratable acidity, and malic acid, for which the order was cultivar > management system > year. PCA on chemical and physico-chemical characteristics of apple skin (Fig 3A, 3B, 3C and 3D) is presented for the first two principal components only, as was the case for pulp. The first PC-score (PC1), which represented 51% of the total variance, had a high contribution of *p*-coumaroylquinic acid, 5-O-caffeoylquinic acid, sucrose, dry matter, and total anthocyanins contents in the positive direction and of glucose and total flavonols in the negative direction (Fig 3A). The second PC-score (PC2) contributed 17% of the total variance and had a high contribution of phloridzin and phloretin-2-O-xyloglucoside in the positive direction (Fig 3A). The pattern was observed for pulp and skin (Figs 2B and 3B). ‘Ariane’ samples were represented in the positive part of PC1 (Fig 3B) and well clustered. Although ‘Melrose’ and Smoothee samples were close, they were well clustered and did not overlap. The clear discrimination between ‘Ariane’ and the other two cultivars was linked to the high anthocyanins concentration in ‘Ariane’. Moreover, ‘Ariane’ also had higher contents of *p*-coumaroylquinic acid, 5-O-caffeoylquinic acid, malic acid, sucrose but lower glucose and total flavonols contents (Fig 3A and S3 Table). Again consistently with ANOVA (Table 4), the inter-annual (Fig 3C) differences were less clearly defined than the cultivar differences. As in the pulp, 2011 and 2013 were fairly well discriminated by PCA forming separated clusters. In contrast, 2012 was distributed in clusters that partly overlapped the 2013 year. As for pulp, since all samples were overlapped, no sample discrimination by management system (Fig 3D) was displayed, unlike cultivar and year.

PCAs and ANOVAs of pulp and skin highlighted cultivar and year as main factors affecting metabolite profiles and fruit characteristics. From these multivariate analyses, the management system appeared to have a lower impact on metabolites profile of fruits than cultivar and year.

### Investigation of the effect of the management system, case study of 2011

To better pinpoint the effect of management system on agronomic variables, and primary and secondary metabolites of apple fruits, an inter-cultivar ANOVA analysis of 2011 year was conducted. The year 2011 was chosen because it permitted a satisfying control of major pests and all three cultivars produced ‘normal’ yield (i.e., no fruit underload due to alternate bearing) (S1 Table).

**Fig 4:**
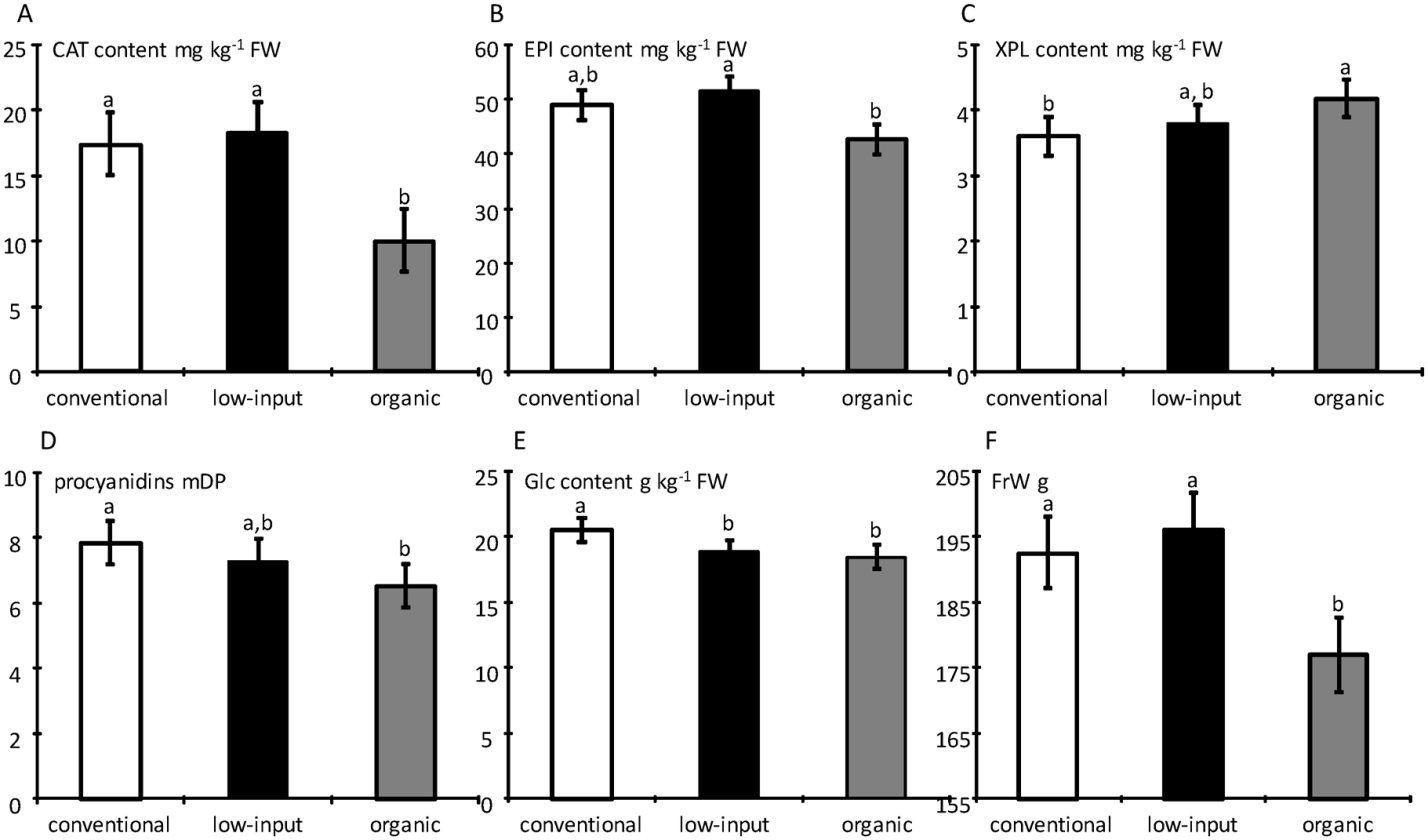
Comparison of metabolite levels in the apple pulp and fruit weight as function of management system for variables with significant management system effects (P ≤ 0.05, Table 5) in 2011. For each variable, values marked with the same letter do not differ significantly (P ≥ 0.05).

In the pulp, the cultivar effect was significant (P < 0.05) for all tested variables, except for phloretin-2-O-xyloglucoside and citric acid (Table 5). The management system was only significant for fruit weight, glucose, (+)-catechin, (-)-epicatechin, mDP and phloretin-2O-xyloglucoside (Table 5). F-values of cultivar effects were higher than F-values of management system except for (+)-catechin and phloretin-2-O-xyloglucoside. Although system management effects were observed, organic fruits did not necessarily contain higher primary or secondary metabolite contents. Fig 4 shows the values per management system for the few metabolites for which management systems effects were significant (Table 5). Organic fruits had significantly lower content of (+)-catechin and (-)-epicatechin compared to conventional and low-input samples. However, for phloretin-2-O-xyloglucoside a significantly higher content was found in organic samples compared to the conventional system. The mDP of procyanidins was significantly lower in organic compared to conventional samples. The same trend was observed for glucose presenting highest concentration in the conventional samples compared to both other management systems that did not differ among them. The fruit weight was similar in the low-input and conventional samples, but was significantly lower in the organic samples.

**Table 5:** Fisher’s F-values associated with two-way ANOVAs (management system and cultivar) performed on apple pulp and skin metabolites, dry matter, and fruit weight in 2011.

	Apple tissues			
	Pulp		Skin	
	Cultivar	Management system	Cultivar	Management system
Fruit Weight	17.9	14.7	-	-
	***	**	-	-
Dry matter	203.7	3.3	87.9	1.2
	***	ns	***	ns
Total soluble solids	23.3	1.5	49.0	1.1
	***	ns	***	ns
Glucose	199.0	6.1	90.4	2.6
	***	**	***	ns
Fructose	6.3	1.0	2.9	0.1
	**	ns	ns	ns
Sucrose	589.7	3.3	252.0	3.3
	***	ns	***	ns
Sorbitol	34.8	0.2	78.5	6.9
	***	ns	***	**
Titratable acidity	197.9	0.6	364.5	5.0
	***	ns	***	*
Malic acid	154.3	1.1	541.2	9.3
	***	ns	***	**
Citric acid	2.3	1.4	11.4	1.4
	ns	ns	**	ns
Total phenolics	26.4	1.1	34.7	2.0
	***	ns	***	ns
Procyanidins	6.3	1.0	52.1	0.6
	**	ns	***	ns
mDP	13.9	4.3	6.0	3.6
	**	*	**	*
(-)-epicatechin	20.8	12.7	19.7	9.1
	***	**	***	**
(+)-catechin	4.1	16.2	12.1	1.8
	*	***	**	ns
Phloridzin	8.3	1.5	1.8	7.4
	**	ns	ns	**
Phloretin-2-O-xyloglucoside	2.7	4.7	2.8	0.9
	ns	*	ns	ns
5CQA	204.1	0.3	1353.9	10.5
	***	ns	***	**
*p*CoQA	62.0	2.1	207.9	0.2
	***	ns	***	ns
Flavonols	-	-	27.5	6.2
	-	-	***	**
Anthocyanins	-	-	202.2	5.7
	-	-	***	*

mDP: average degree of polymerization of procyanidins, 5CQA: 5-O-caffeoylquinic acid, pCoQA: p-coumaroylquinic acid, ns: non significant,

*: significant at P ≤0.05

**: significant at P ≤0.01

***: significant at P ≤0.001

**Fig 5:**
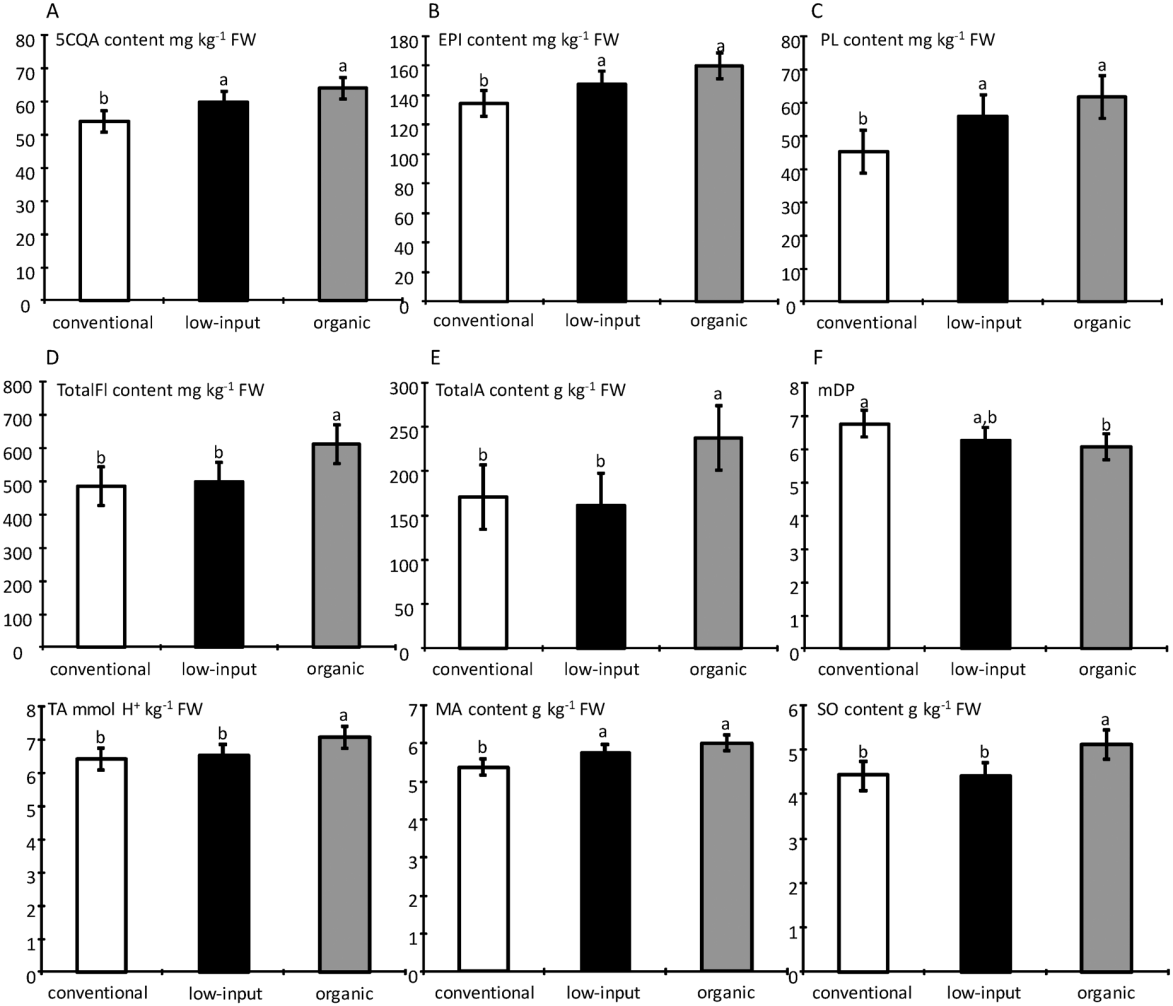
Comparison of metabolite levels in the apple skin as function of the management system for variables with significant management system effects (P ≤ 0.05, Table 5) in 2011. For each variable, values marked with the same letter do not differ significantly (P ≥ 0.05).

In the skin, the cultivar effect was significant (P < 0.05) for all variables tested, except for phloretin-2-O-xyloglucoside, phloridzin and fructose (Table 5). The management system was significant for 5-O-caffeoylquinic acid, malic acid, (-)-epicatechin, phloridzin, sorbitol, total flavonols, total anthocyanins, titratable acidity and mDP (Table 5, Fig 5). F-values of cultivar effects were also higher than F-values of management system ones except for phloridzin. Conventional samples had significantly lower skin content of 5-O-caffeoylquinic acid (Fig 5A), (-)-epicatechin (Fig 5B), phloridzin (Fig 5C), and malic acid (Fig 5H) compared to organic and low-input samples, for which concentrations were similar. Similarly, a significantly lower content of total flavonols, total anthocyanins, sorbitol and titratable acidity were found in the skin of conventional and low-input samples compared to organic samples. However, the mDP of procyanidins was significantly lower in organic samples compared to the conventional samples.

Differences in fruit composition between the management systems were very limited, since they affected only few minor phenolics and with limited differences. In addition, within the same class of phenolics (e.g. (-)-epicatechin *vs* (+)-catechin and phloridzin *vs* phloretin-2-O-xyloglucoside in the skin), the differences observed for one of the compounds were not found for the other although these metabolites share the same biosynthetic pathway, making it difficult to generalize the management system effect. In the skin, a trend towards higher or equal phenolic contents was found for organic samples compared to the two other management systems. In contrast, in the pulp, the levels were similar between systems except for flavan-3-ols monomers ((-)-epicatechin and (+)-catechin). In fact, organic samples had a lower content of flavan-3-ol monomers compared to conventional and low-input samples.

### Effect of management system on total phenolics content during the three years

The stability of the management system effect between the years was studied using the total phenolic content of apple pulp and skin.

**Fig 6:**
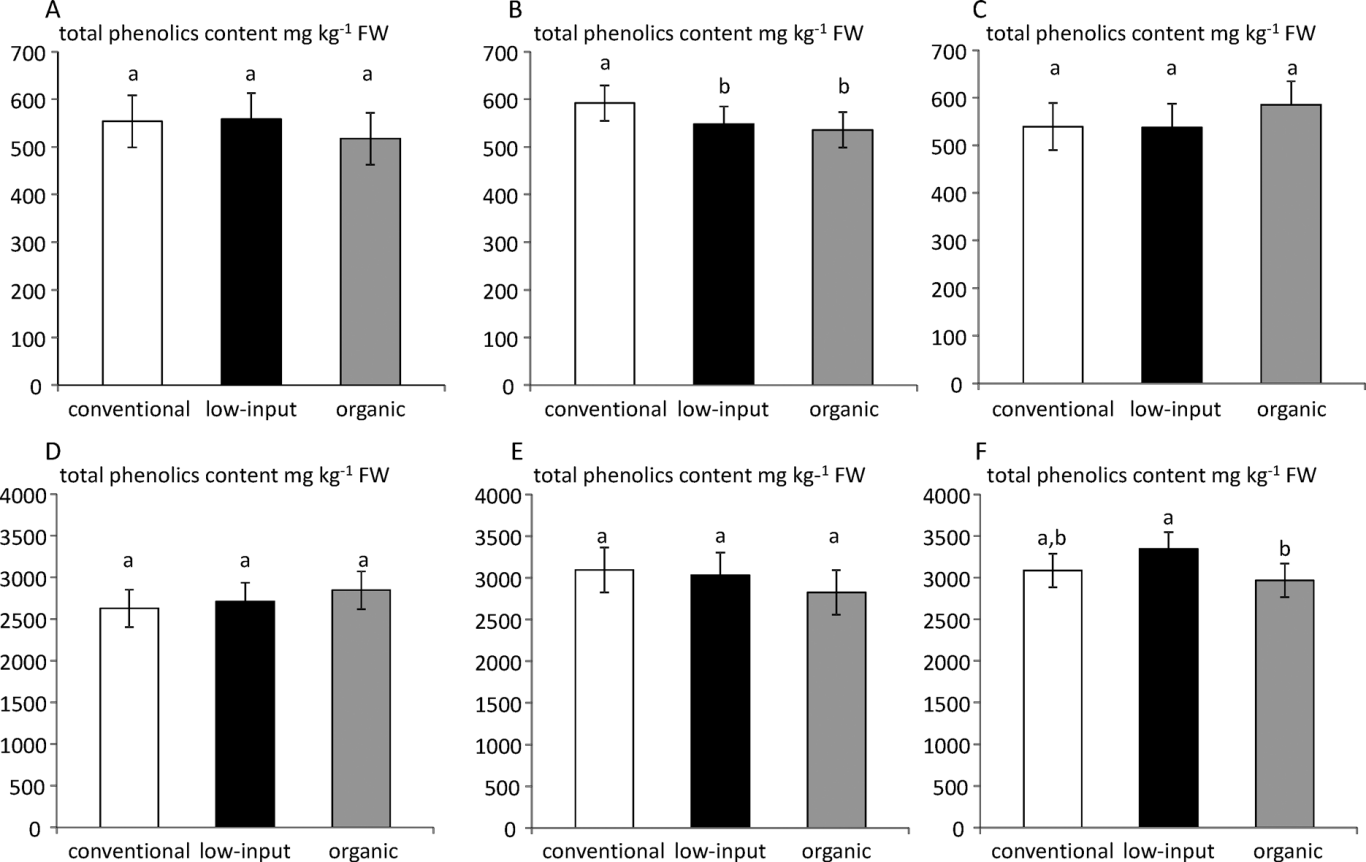
Sum of total phenolics content in apple pulp for the years 2011 (A), 2012 (B), and 2013 (C) and in apple skin for 2011 (D), 2012 (E), 2013 (F) for fruit grown under conventional, low-input and organic management systems. Values are means of three different samples analyzed independently.

In 2011, the total phenolics content of apple pulp (Fig 6A) did not significantly differ between the three management systems. In 2012 (Fig 6B), a significant higher level was observed in conventionally grown apples compared to organic and low-input systems. In contrast, in 2013 (Fig 6C) the total phenolics content tended to be higher in the organic system, but the difference was not significant.

In 2011, the total phenolics content of apple skin (Fig 6D) did not significantly differ between the three management systems although a slightly higher level was observed in the organic system. In 2012 (Fig 6E), the opposite was observed, the total phenolics content tended to be higher in the conventional system, but the difference was not significant. Finally, in 2013 (Fig 6F), only the effect of the cultivation system was significant. The total phenolics content of apple skin tended to be higher in low-input system compared to the organic system, the conventional system being intermediate.

## Discussion

It is very difficult to find data on the composition of fruit comparing the management systems without potential artefacts due to differences in pedoclimatic conditions, orcharding practices or even cultivar. Our aim was therefore to assess the influence of the management system, independently of other potential confounding effects, on fruit attributes, primary and secondary metabolites in apples over several years and from different cultivars. The overall fruit quality was evaluated in apples grown under well-defined organic, low-input and conventional management systems, located in the same system experiment, and planted with three cultivars differing in scab susceptibility: ‘Ariane’ (Vf-resistant), ‘Melrose’ (low-susceptibility) and ‘Smoothee’ (susceptible) for three years.

There were clear differences between the management practices systems, especially in terms of pest pressure and yield which were respectively the highest and the lowest in organic systems [34]. This may be related to management constraints (e.g., no direct measure, less efficient inputs in organic) and/or practices (organic fruit load was adjusted at lower levels to avoid alternate bearing). In contrast, the total nitrogen supply was very close for all three systems though, of course, the nature of the supply -and therefore its availability for trees- may differ.

The cultivars had medium to large fruit compared to data of [24, 37]. Dry matter variation was in accordance with previous studies [23, 37]. The first systematic difference observed here was that the organic orchard management produced fruits of lower fresh weight than low-input and conventional orchard management systems, despite a lower targeted crop load. This may be due to tree nutrition (e.g. lower nitrogen availability at some periods) and pest infestation, especially by the rosy apple aphid which is known to alter fruit growth [38]. In the organic orchard, lower nitrogen availability could reduce fruit cell divisions leading to fewer cells per fruit and smaller fruits [39]. Some authors [33, 40] also found lower fruit weight for apples produced under organic orchards management, probably due to smaller cells and less intercellular spaces [40].

A global technique -mid-infrared spectroscopy- performed directly on apple skin and pulp homogenates did not discriminate samples according to the systems of cultivation. Samples discrimination was mainly function of the cultivar, the year effect was visible though less pronounced (some overlapping). The effect of management systems appeared lower than the genetic and annual effects.

Among primary metabolites, total soluble solids, titratable acidity, four sugars and two organics acids were determined. All concentrations and relative composition are consistent with previous studies, which have shown a wide variation in the total soluble solid, titratable acidy and individual sugars and organic acids content depending on the cultivar [20, 23–24, 41]. In the present study, on the basis of the year 2011, only malic acid, sorbitol and titratable acidity in the skin were significantly higher in apple produced under organic management compared to conventional. However, [42] found that apples from integrated management practices, that corresponds to our low-input management system, contained higher levels of total sugars, as well as organic acids than organically produced apples. Conversely, some authors showed no substantial differences between apples from organic and integrated orchards in terms of fruit quality at harvest (sugar and acid contents, soluble solids content) [43]. Others also compared organic and conventional apple production and did not find any differences in the titratable acidity and total soluble solid contents [33]. From literature and the present results, it seems that the management system does not consistently affect the fruit primary metabolites.

The phenolic contents were in the range of previous work on dessert apple which quantified all five classes of phenolic in the pulp (177–1596 mg kg-1 FW; [14, 18–19, 21]) as well as in the skin (1016–7658 mg kg-1 FW; [14, 18, 19, 21, 30]). All concentrations and relative composition of each class are consistent with literature. Previous studies have also shown a wide variation in the phenolic content depending on the cultivar [18, 19, 21–22, 25, 44, 45]. These different works deal with the characterization of phenolic profiles of dessert apples [18, 19, 25], of apple genotypes selected for processing with high phenolic content [21] without reaching however extreme concentrations known in cider apples [18, 44, 45]. For secondary metabolites, depending to the compounds, the management system effect could be significant in some cases of the present study, but still much lower than the cultivar and year effects. Furthermore, when an effect was highlighted, management systems ranked differently depending on the compounds and/or the fruit tissues (skin or pulp). No general trend could be observed between systems, and the total phenolics content did not differ between systems except for pulp in 2012. Furthermore, within the same phenolic class, an effect could be observed on one compound but not on another, making it difficult to generalize the system effect on a particular biosynthetic pathway. If the present results were expressed per fruit, the conclusions would be the same as observed for the pulp (data not shown) because pulp prevails in the total weight of the fruit. In contrast to our study, some authors found for the year 2005 a higher content of phenolics in organically grown compared to conventionally grown apples [7], but the crops were grown at two different farms, which could have induced a variation not related to management system, even though the farms had relatively close geographical locations. Similarly, another study described higher contents of phenolics in the pulp of organically grown apples compared to apples from integrated production [46], but this study is difficult to interpret with regards to ours because different cultivars were compared in the organic and integrated cropping systems, which does not permit to disentangle the respective effects of the production system *per se* and the genotype. Of course, we are aware that the cultivar is part of the orchard, and as some cultivars are less easy to manage under organic or low-input farming, apple cultivars available on the market are also related to the production system, but this point is beyond the scope of this work. Recent studies of organic and conventional apples by [30] and [8] support our finding. The differences observed by some authors (i.e., more polyphenols in organic management system compared to conventional) can be explained by different environmental conditions between management systems, differential responses related to the cultivar, and/or the year climate. They may also be due to management systems more contrasted than ours. In our case, most of the differences between systems, and above all between organic and both other systems, were related to crop protection and fertilizing whereas other practices were similar ([34]; Table 1). All systems had especially a similar management for water with no water stress which is known to induce differences in phenolics [47]. Furthermore, the levels of nitrogen fertilization were moderate and similar in the three management systems, in spite of different types of input. It should be noted that these factors (irrigation, levels of nitrogen) are independent of the organic *versus* conventional denomination. This sends back to the need to precisely document the practices carried out in the study orchards. Moreover, the biosynthetic pathways of primary and secondary metabolites are complex and multiple factors may affect apple composition.

Finally, three management systems (i.e., organic, low-inputs and conventional) instead of generally two systems were studied in a well-documented system experiment, displaying a range of cultural practices rather than chemical/non chemical archetypes, and eliminating the bias induced by uncontrolled environmental external factors. Under these controlled growing conditions, cultivar and year-to-year effects were by far the most effective in determining the quantitative primary and secondary metabolites composition of apples, while the management system (organic, low-input or conventional) had little and no consistent influence on fruit composition. More broadly, all compounds of the apple contribute to its health value and its taste, and other quality criteria (e.g., possible fruit pesticide residues) can also influence the choices of consumers in buying ‘eco-friendly’ or organic apples [48] beside more general considerations (e.g., environmental impact of cropping practices).

## Supporting Information

S1 Table Agronomic performances of Ariane in the three management systems in 2011. (DOCX)

S2 Table Mean fruit weight (g), dry matter (%), and concentration of phenolics (mg kg-1 FW), individual sugars (g kg-1 FW), organic acids (g kg-1 FW), titratable acidity (mmol H+ kg-1 FW), total soluble solid contents (°Brix) of apple pulp cultivars of samples from 3 management systems x 3 cultivars in 2011 (A), 2012 (B), and 2013 (C). (DOCX)

S3 Table Mean dry matter (%), and concentration of various phenolics (mg kg-1 FW), individual sugars (g kg-1 FW), organic acids (g kg-1 FW), titratable acidity (mmol H+ kg-1 FW), total soluble solid contents (°Brix) of apple skin cultivars of samples from 3 management systems x 3 cultivars in 2011 (A), 2012 (B), and 2013 (C). (DOCX)
