# Supplementary material for: Cultivar and Year Rather than Agricultural Practices Affect Primary and Secondary Metabolites in Apple Fruit
Source: PLoS One. 2015 Nov 30;10(11):e0141916. doi: 10.1371/journal.pone.0141916 (PMC4664253; doi:10.1371/journal.pone.0141916)
Supplement: S1 Table — (DOCX) [file pone.0141916.s001.docx]

**S1 Table. Agronomic performances of Ariane in the three management systems in 2011.**

| Management system | Conventional | Low-input | Organic |
| --- | --- | --- | --- |
| Total yield (t ha^-1^) | 39.5 | 38.4 | 22.4 |
| Fruit diameter of prevailing (> 25% of the total weight) caliber classes^a^ | 70/75 and 75/80 | 70/75 and 75/80 | 70/75 |
| Fruit average weight in the prevailing caliber(s) (g) | 165 | 159 | 142 |
| trunk cross section area (TCSA) (cm²) | 38.70 | 43.96 | 38.10 |
| Pesticide use (yearly number of registered applied doses):  -Total  -Total excluding biocontrol^b^ products | 32.0  26.0 | 25.5  16.8 | 23.4  14.7 |
| Fruit damage at harvest (% total fruit):  -total  -scab damage  - aphid damage  -codling moth (Tortricidae: *Cydia pomonella*) damage | 1.0  0.0 (scab resistant)  0.1  0.9 | 1.7  0.0 (scab resistant) 0.5  1.2 | 5.1  0.0 (scab resistant) 3.0  2.1 |

^a^Difference in diameter between fruit in the same caliber limited to 5 mm and categories of class I and Extra fruit ranging from 60 to 100 mm.

^b^Granulosis virus and nematodes applications against codling moth, and kaolin applications against aphids are counted as biocontrol products whereas sulphur, copper and mineral oils mainly applied in organic farming are not distinguished in the total.
