# Supplementary material for: Cultivar and Year Rather than Agricultural Practices Affect Primary and Secondary Metabolites in Apple Fruit
Source: PLoS One. 2015 Nov 30;10(11):e0141916. doi: 10.1371/journal.pone.0141916 (PMC4664253; doi:10.1371/journal.pone.0141916)
Supplement: S2 Table — (DOCX) [file pone.0141916.s002.docx]

**S2 Table. Mean fruit weight (g), dry matter (%), and concentration of phenolics (mg kg^-1^ FW), individual sugars (g kg^-1^ FW), organic acids (g kg^-1^ FW), titratable acidity (mmol H^+^ kg^-1^ FW), total soluble solid contents (°Brix) of apple pulp cultivars of samples from 3 management systems x 3 cultivars in 2011 (A), 2012 (B), and 2013 (C).** FrW: Fruit Weight, DM: Dry Mater, TSS: Total Soluble Solids, SUC: sucrose, Glc: glucose, FRU: Fructose, SO: Sorbitol, TA: Titratable Acidity, MA: Malic Acid, CA: Citric Acid, CA : (+)-catechin, EPI: (-)-epicatechin, PC: procyanidins, mDP: average degree of polymerization of procyanidins, XPL: phloretin-2-O-xyloglucoside, PL: phloridzin, 5CQA: 5-O-caffeoylquinic acid, *p*CoQA : *p-*coumaroylquinic acid, Tot: sum of phenolic, SD: pool standard deviation, nd: not detected.

|  | FrW | DM | TSS | SUC | Glc | FRU | SO | TA | MA | CA | CAT | EPI | PC | mDP | XPL | PL | 5CQA | *p*CoQA | Tot |
| --- | --- | --- | --- | --- | --- | --- | --- | --- | --- | --- | --- | --- | --- | --- | --- | --- | --- | --- | --- |
| 1. 2011 | | | | | | | | | | | | | | | | | | | |
| **Ariane** |  |  |  |  |  |  |  |  |  |  |  |  |  |  |  |  |  |  |  |
| conventional | 188.7 | 17.1 | 15.4 | 47.9 | 14.7 | 63.2 | 7.0 | 11.0 | 8.5 | nd | 22.7 | 60.6 | 408.0 | 7.1 | 3.8 | 11.8 | 153.2 | 6.7 | 666.8 |
| low-inputs | 179.1 | 17.0 | 15.1 | 43.5 | 12.5 | 63.8 | 5.8 | 10.5 | 8.5 | nd | 21.9 | 55.6 | 427.4 | 7.0 | 3.8 | 11.2 | 153.6 | 6.6 | 680.1 |
| organic | 172.6 | 17.4 | 14.1 | 44.3 | 12.9 | 62.2 | 6.3 | 11.6 | 9.4 | nd | 8.0 | 47.2 | 414.1 | 5.1 | 4.7 | 15.3 | 162.3 | 5.9 | 657.4 |
| **Melrose** |  |  |  |  |  |  |  |  |  |  |  |  |  |  |  |  |  |  |  |
| conventional | 201.3 | 13.4 | 12.7 | 12.3 | 26.4 | 66.5 | 2.7 | 7.1 | 5.3 | 0.02 | 13.7 | 41.3 | 390.3 | 7.2 | 3.5 | 6.9 | 46.8 | 3.4 | 505.9 |
| low-inputs | 224.9 | 13.2 | 11.7 | 10.8 | 25.0 | 66.6 | 2.7 | 7.0 | 5.8 | 0.00 | 16.7 | 51.1 | 350.1 | 6.6 | 4.0 | 7.6 | 50.5 | 4.4 | 484.7 |
| organic | 177.8 | 13.9 | 12.7 | 10.5 | 25.2 | 68.3 | 2.7 | 6.2 | 4.7 | nd | 8.6 | 38.9 | 289.1 | 6.3 | 3.9 | 8.9 | 48.1 | 3.3 | 400.7 |
| **Smoothee** |  |  |  |  |  |  |  |  |  |  |  |  |  |  |  |  |  |  |  |
| conventional | 187.8 | 15.4 | 13.6 | 23.8 | 20.3 | 67.5 | 2.5 | 6.0 | 4.9 | nd | 15.7 | 44.8 | 342.8 | 9.3 | 3.4 | 9.2 | 70.6 | 2.8 | 489.5 |
| low-input | 184.5 | 15.5 | 13.1 | 22.3 | 19.2 | 64.2 | 3.0 | 6.0 | 5.0 | nd | 16.1 | 47.9 | 353.2 | 8.3 | 3.6 | 10.0 | 76.4 | 3.2 | 510.4 |
| organic | 180.5 | 15.7 | 13.6 | 27.5 | 17.2 | 69.4 | 2.3 | 6.6 | 5.6 | nd | 13.5 | 41.8 | 349.7 | 8.2 | 3.9 | 9.7 | 72.4 | 3.1 | 494.1 |
| 1. 2012 | | | | | | | | | | | | | | | | | | | |
| **Ariane** |  |  |  |  |  |  |  |  |  |  |  |  |  |  |  |  |  |  |  |
| conventional | 176.4 | 17.1 | 13.9 | 49.9 | 10.5 | 60.0 | 7.1 | 9.6 | 7.4 | 0.15 | 14.9 | 38.9 | 333.9 | 4.4 | 3.6 | 11.9 | 160.9 | 7.9 | 572.0 |
| low-input | 181.1 | 17.8 | 14.6 | 50.0 | 11.8 | 70.3 | 8.7 | 10.1 | 8.8 | 0.47 | 13.5 | 38.9 | 369.2 | 5.1 | 4.4 | 14.1 | 170.3 | 6.8 | 617.1 |
| organic | 175.9 | 18.8 | 15.5 | 53.9 | 13.4 | 70.3 | 11.4 | 11.0 | 9.8 | 0.46 | 11.4 | 34.1 | 381.4 | 5.1 | 5.3 | 17.3 | 185.6 | 7.5 | 642.6 |
| **Melrose** |  |  |  |  |  |  |  |  |  |  |  |  |  |  |  |  |  |  |  |
| conventional | 178.4 | 13.3 | 10.8 | 19.8 | 23.3 | 70.5 | 2.0 | 6.4 | 5.1 | 0.19 | 14.3 | 50.0 | 500.7 | 4.8 | 6.1 | 10.7 | 77.8 | 8.7 | 668.2 |
| low-input | 188.1 | 14.6 | 11.5 | 24.3 | 22.1 | 70.8 | 2.6 | 6.9 | 5.3 | 0.21 | 15.2 | 56.5 | 377.5 | 6.0 | 5.3 | 10.2 | 69.1 | 5.9 | 539.7 |
| organic | 184.9 | 14.3 | 11.4 | 22.2 | 21.6 | 69.6 | 2.3 | 7.4 | 5.8 | 0.27 | 12.0 | 58.1 | 329.9 | 6.7 | 5.0 | 9.7 | 57.0 | 5.8 | 477.3 |
| **Smoothee** |  |  |  |  |  |  |  |  |  |  |  |  |  |  |  |  |  |  |  |
| conventional | 216.4 | 16.7 | 12.8 | 26.8 | 18.5 | 6.0 | 2.2 | 6.7 | 5.3 | 0.05 | 11.8 | 37.0 | 368.1 | 4.2 | 4.8 | 13.4 | 96.6 | 4.1 | 538.8 |
| low-input | 223.8 | 16.2 | 12.4 | 27.9 | 16.2 | 57.7 | 1.9 | 7.1 | 5.9 | nd | 11.5 | 35.9 | 334.1 | 4.3 | 4.1 | 12.1 | 86.4 | 3.7 | 487.8 |
| organic | 154.7 | 15.7 | 12.1 | 20.8 | 20.6 | 57.5 | 1.9 | 7.5 | 5.9 | 0.21 | 11.1 | 36.3 | 337.1 | 4.3 | 5.3 | 13.0 | 21.2 | 4.0 | 487.9 |
| (C°) 2013 | | | | | | | | | | | | | | | | | | | |
| **Ariane** |  |  |  |  |  |  |  |  |  |  |  |  |  |  |  |  |  |  |  |
| conventional | 136.7 | 16.7 | 13.6 | 56.4 | 9.6 | 61.1 | 5.3 | 10.6 | 7.9 | nd | 16.9 | 50.0 | 350.3 | 5.7 | 4.4 | 15.3 | 191.6 | 6.0 | 634.5 |
| low-input | 136.3 | 16.6 | 13.8 | 64.9 | 11.2 | 53.3 | 6.8 | 10.1 | 7.6 | 0.03 | 17.3 | 54.7 | 361.4 | 5.3 | 4.4 | 15.4 | 185.4 | 6.0 | 644.6 |
| organic | 131.7 | 17.7 | 14.5 | 63.4 | 11.9 | 71.4 | 6.0 | 10.6 | 8.4 | 0.06 | 17.2 | 49.9 | 414.3 | 5.9 | 4.9 | 20.2 | 214.7 | 6.5 | 727.7 |
| **Melrose** |  |  |  |  |  |  |  |  |  |  |  |  |  |  |  |  |  |  |  |
| conventional | 214.6 | 13.3 | 12.2 | 39.8 | 22.4 | 73.3 | 2.4 | 7.0 | 3.8 | nd | 17.9 | 55.8 | 330.4 | 6.1 | 5.6 | 9.1 | 82.3 | 4.5 | 503.6 |
| low-input | 216.0 | 13.3 | 12.4 | 50.4 | 18.3 | 77.7 | 2.5 | 6.7 | 4.4 | nd | 18.5 | 64.3 | 339.2 | 6.0 | 5.6 | 9.1 | 82.3 | 4.9 | 523.9 |
| organic | 171.0 | 13.9 | 11.5 | 50.3 | 17.5 | 90.6 | 2.5 | 7.6 | 4.1 | 0.09 | 16.2 | 39.1 | 304.4 | 6.6 | 7.0 | 14.2 | 84.0 | 4.6 | 469.5 |
| **Smoothee** |  |  |  |  |  |  |  |  |  |  |  |  |  |  |  |  |  |  |  |
| conventional | 157.8 | 14.9 | 12.0 | 34.1 | 22.2 | 69.8 | 1.6 | 6.6 | 5.1 | 0.03 | 14.2 | 48.8 | 289.3 | 6.1 | 5.0 | 14.4 | 93.8 | 4.5 | 478.9 |
| low-input | 177.4 | 15.6 | 12.5 | 38.8 | 18.9 | 78.1 | 1.9 | 6.7 | 5.0 | nd | 12.7 | 42.3 | 257.5 | 5.9 | 4.9 | 14.1 | 113.8 | 4.5 | 444.9 |
| organic | 148.9 | 15.5 | 12.4 | 45.7 | 16.6 | 68.0 | 1.7 | 7.1 | 5.8 | nd | 13.6 | 46.9 | 370.2 | 7.7 | 5.1 | 13.3 | 103.6 | 4.0 | 556.8 |
| SD | *25.5* | *1.6* | *1.3* | *16.3* | *5.0* | *8.9* | *2.6* | *1.9* | *1.7* | *nd* | *4.0* | *10.3* | *52.1* | *1.6* | *0.9* | *3.4* | *50.7* | *1.6* | *94.4* |
