# Supplementary material for: Cultivar and Year Rather than Agricultural Practices Affect Primary and Secondary Metabolites in Apple Fruit
Source: PLoS One. 2015 Nov 30;10(11):e0141916. doi: 10.1371/journal.pone.0141916 (PMC4664253; doi:10.1371/journal.pone.0141916)
Supplement: S3 Table — (DOCX) [file pone.0141916.s003.docx]

**S3 Table. Mean dry matter (%), and concentration of various phenolics (mg kg^-1^ FW), individual sugars (g kg^-1^ FW), organic acids (g kg^-1^ FW), titratable acidity (mmol H^+^ kg^-1^ FW), total soluble solid contents (°Brix) of apple skin cultivars of samples from 3 management systems x 3 cultivars in 2011 (A), 2012 (B), and 2013 (C).** DM: Dry Mater, TSS: Total Soluble Solids, SUC: sucrose, Glc: glucose, FRU: Fructose, SO: Sorbitol, TA: Titratable Acidity, MA: Malic Acid, CA: Citric Acid, CAT: (+)-catechin, EPI: (-)-epicatechin, PC: procyanidins, mDP: average degree of polymerization of procyanidins, XPL: phloretin-2-O-xyloglucoside, PL: phloridzin, 5CQA: 5-O-caffeoylquinic acid, *p*CoQA: *p*-coumaroylquinic acid, TotalFl: sum of flavonols, TotalA: sum of anthocyanins, Tot: sum of phenolics, SD: pool standard deviation.

|  | DM | TSS | SUC | Glc | FRU | SO | TA | MA | CA | CAT | EPI | PC | mDP | XPL | PL | 5CQA | *p*CoQA | | TotalFl | TotalA | Tot |
| --- | --- | --- | --- | --- | --- | --- | --- | --- | --- | --- | --- | --- | --- | --- | --- | --- | --- | --- | --- | --- | --- |
| 1. 2011 | | | | | | | | | | | | | | | | | | | | | |
| **Ariane** |  |  |  |  |  |  |  |  |  |  |  |  |  |  |  |  | |  |  |  |  |
| conventional | 22.3 | 16.0 | 47.1 | 17.8 | 57.5 | 6.6 | 9.5 | 7.8 | nd | 20.9 | 153.5 | 2098.8 | 6.1 | 16.4 | 32.0 | 112.3 | | 4.8 | 373.8 | 327.4 | 3139.9 |
| low-input | 21.6 | 15.0 | 41.8 | 17.6 | 60.0 | 5.9 | 9.1 | 8.1 | nd | 12.7 | 148.4 | 2011.5 | 6.1 | 22.0 | 51.7 | 117.0 | | 4.2 | 350.6 | 303.7 | 3021.9 |
| organic | 2.8 | 15.4 | 43.8 | 16.4 | 56.3 | 6.2 | 11.3 | 9.0 | nd | 21.0 | 199.5 | 2318.8 | 6.0 | 21.0 | 65.6 | 136.8 | | 4.5 | 379.5 | 401.7 | 3548.5 |
| **Melrose** |  |  |  |  |  |  |  |  |  |  |  |  |  |  |  |  | |  |  |  |  |
| conventional | 18.5 | 12.2 | 17.2 | 26.2 | 58.0 | 3.6 | 4.2 | 3.5 | nd | 12.0 | 138.3 | 1509.5 | 7.7 | 21.6 | 51.3 | 12.4 | | 1.0 | 524.6 | 184.1 | 2545.8 |
| low-input | 19.0 | 12.2 | 16.0 | 23.9 | 56.2 | 3.2 | 4.6 | 3.9 | nd | 12.3 | 152.5 | 1745.4 | 6.7 | 26.1 | 61.8 | 14.6 | | 1.5 | 541.4 | 180.5 | 2736.0 |
| organic | 18.6 | 12.6 | 20.3 | 26.5 | 64.4 | 4.5 | 3.6 | 3.3 | nd | 12.8 | 140.6 | 1315.7 | 6.3 | 22.4 | 60.4 | 17.5 | | 1.4 | 656.4 | 309.8 | 2540.3 |
| **Smoothee** |  |  |  |  |  |  |  |  |  |  |  |  |  |  |  |  | |  |  |  |  |
| conventional | 20.6 | 13.3 | 22.6 | 24.6 | 56.3 | 3.1 | 5.4 | 4.9 | nd | 9.9 | 110.8 | 1498.3 | 6.5 | 19.2 | 52.4 | 37.4 | | 2.0 | 556.1 | nd | 2286.1 |
| low-input | 20.5 | 13.3 | 24.0 | 22.7 | 53.3 | 4.1 | 5.9 | 5.3 | nd | 11.6 | 140.9 | 1501.1 | 6.0 | 18.3 | 54.1 | 47.5 | | 2.0 | 600.5 | nd | 2375.9 |
| organic | 20.9 | 13.7 | 27.2 | 23.9 | 52.2 | 4.7 | 6.3 | 5.7 | nd | 11.4 | 139.1 | 1388.9 | 5.9 | 15.1 | 59.2 | 37.3 | | 2.1 | 791.6 | nd | 2444.7 |
| 1. 2012 | | | | | | | | | | | | | | | | | | | | | |
| **Ariane** |  |  |  |  |  |  |  |  |  |  |  |  |  |  |  |  | |  |  |  |  |
| conventional | 22.6 | 15.9 | 40.9 | 12.9 | 50.7 | 6.5 | 9.7 | 6.7 | 0.19 | 35.6 | 204.1 | 2261.5 | 6.1 | 25.4 | 68.2 | 98.9 | | 6.0 | 335.1 | 257.3 | 3292.0 |
| low-input | 23.9 | 16.5 | 40.7 | 13.4 | 45.7 | 8.2 | 10.1 | 7.1 | 0.36 | 36.4 | 204.1 | 2346.7 | 6.2 | 36.9 | 70.2 | 115.7 | | 6.1 | 328.1 | 281.8 | 3425.9 |
| organic | 24.6 | 18.7 | 46.3 | 12.9 | 46.5 | 10.2 | 11.5 | 8.0 | 0.36 | 26.8 | 201.1 | 2099.8 | 6.1 | 34.7 | 76.1 | 124.0 | | 6.2 | 318.4 | 253.3 | 3140.4 |
| **Melrose** |  |  |  |  |  |  |  |  |  |  |  |  |  |  |  |  | |  |  |  |  |
| conventional | 19.7 | 11.2 | 19.7 | 21.2 | 53.3 | 2.9 | 4.3 | 3.9 | 0.20 | 21.6 | 201.6 | 2502.5 | 6.7 | 50.6 | 103.2 | 27.1 | | 2.9 | 462.1 | 45.2 | 3461.9 |
| low-input | 21.5 | 12.0 | 23.8 | 21.0 | 54.2 | 3.7 | 4.4 | 4.0 | 0.22 | 17.7 | 225.4 | 2311.4 | 6.6 | 45.1 | 104.8 | 23.7 | | 2.4 | 578.7 | 107.2 | 3416.3 |
| organic | 20.5 | 11.6 | 22.5 | 19.2 | 59.0 | 3.6 | 4.2 | 4.4 | 0.24 | 18.2 | 206.8 | 2080.5 | 5.9 | 41.6 | 89.1 | 17.2 | | 2.0 | 472.4 | 74.8 | 3079.5 |
| **Smoothee** |  |  |  |  |  |  |  |  |  |  |  |  |  |  |  |  | |  |  |  |  |
| conventional | 22.5 | 13.0 | 22.4 | 23.0 | 44.4 | 3.0 | 6.3 | 5.5 | nd | 16.6 | 159.1 | 1799.1 | 5.9 | 33.4 | 64.5 | 62.3 | | 4.4 | 392.6 | nd | 2532.0 |
| low-input | 21.6 | 12.6 | 25.2 | 19.8 | 49.6 | 2.7 | 6.7 | 5.8 | nd | 14.7 | 156.1 | 1589.6 | 5.6 | 33.2 | 63.6 | 52.0 | | 2.6 | 347.4 | nd | 2259.2 |
| organic | 21.0 | 12.5 | 19.1 | 24.6 | 49.1 | 3.0 | 6.5 | 6.0 | nd | 17.1 | 130.5 | 1526.2 | 5.2 | 21.2 | 88.5 | 60.4 | | 3.5 | 407.3 | nd | 2254.7 |
| (C°) 2013 | | | | | | | | | | | | | | | | | | | | | |
| **Ariane** |  |  |  |  |  |  |  |  |  |  |  |  |  |  |  |  | |  |  |  |  |
| conventional | 21.8 | 13.8 | 49.8 | 12.1 | 48.9 | 5.9 | 9.6 | 7.3 | 0.07 | 32.3 | 233.7 | 2224.4 | 6.4 | 26.3 | 68.6 | 161.6 | | 5.6 | 293.8 | 270.7 | 3317.1 |
| low-input | 22.2 | 14.2 | 47.5 | 15.7 | 54.7 | 5.7 | 9.6 | 7.8 | nd | 38.6 | 260.1 | 2579.1 | 6.3 | 29.4 | 82.9 | 174.4 | | 6.1 | 394.3 | 322.7 | 3887.6 |
| organic | 24.2 | 15.8 | 51.5 | 13.5 | 53.6 | 7.5 | 10.7 | 8.3 | 0.08 | 30.3 | 206.4 | 2120.1 | 6.5 | 26.9 | 82.0 | 172.8 | | 5.6 | 325.4 | 267.5 | 3237.2 |
| **Melrose** |  |  |  |  |  |  |  |  |  |  |  |  |  |  |  |  | |  |  |  |  |
| conventional | 19.9 | 12.5 | 30.1 | 22.7 | 65.4 | 2.5 | 3.8 | 3.2 | nd | 23.9 | 154.1 | 2041.8 | 6.9 | 28.0 | 87.4 | 49.3 | | 1.9 | 356.4 | 119.4 | 2981.6 |
| low-input | 20.1 | 12.3 | 33.3 | 22.8 | 62.2 | 2.2 | 3.7 | 3.1 | nd | 31.8 | 202.8 | 2188.0 | 7.3 | 30.7 | 91.0 | 54.4 | | 2.3 | 437.3 | 123.7 | 3285.7 |
| organic | 19.9 | 13.0 | 27.1 | 26.3 | 40.3 | 3.5 | 4.3 | 4.2 | 0.02 | 27.4 | 157.2 | 1784.2 | 8.1 | 27.8 | 109.4 | 59.0 | | 2.0 | 480.9 | 143.6 | 2791.5 |
| **Smoothee** |  |  |  |  |  |  |  |  |  |  |  |  |  |  |  |  | |  |  |  |  |
| conventional | 19.8 | 12.5 | 17.7 | 24.6 | 52.9 | 2.5 | 5.8 | 5.0 | 0.02 | 28.3 | 155.2 | 1988.9 | 7.8 | 29.0 | 88.1 | 105.8 | | 3.7 | 559.2 | nd | 2958.2 |
| low-input | 20.2 | 12.7 | 26.0 | 19.9 | 46.7 | 2.7 | 5.7 | 4.7 | nd | 26.7 | 149.7 | 1937.2 | 7.4 | 31.1 | 83.5 | 110.5 | | 2.9 | 509.1 | nd | 2850.6 |
| organic | 20.3 | 12.7 | 33.6 | 20.6 | 62.9 | 3.5 | 6.2 | 5.4 | nd | 24.8 | 146.3 | 1912.5 | 7.6 | 30.0 | 95.1 | 110.2 | | 3.3 | 547.1 | nd | 2870.3 |
| SD | *1.6* | *1.9* | *11.6* | *4.6* | *7.6* | *2.0* | *2.6* | *1.8* | *nd* | *8.8* | *38.8* | *376.9* | *0.8* | *9.3* | *20.3* | *50.3* | | *1.7* | *138.6* | *136.0* | *490.8* |
